# Novel mechanism of drug resistance triggered by tumor-associated macrophages through Heat Shock Factor-1 activation

**DOI:** 10.1007/s00262-023-03612-2

**Published:** 2024-01-27

**Authors:** Alina D. Nikotina, Snezhana A. Vladimirova, Nadezhda E. Kokoreva, Valeria A. Nevdakha, Vladimir F. Lazarev, Liubov S. Kuznetcova, Elena Y. Komarova, Roman V. Suezov, Sergei Efremov, Elizaveta Leonova, Viktor G. Kartsev, Nikolay D. Aksenov, Boris A. Margulis, Irina V. Guzhova

**Affiliations:** 1 Laboratory of Cell Protection Mechanisms, Institute of Cytology of Russian Academy of Sciences, St. Petersburg, 194064 Russia; 2 Saint-Petersburg State University Hospital, 190103 St. Petersburg, Russia; 3 InterBioScreen, Chernogolovka, Moscow, 142432 Russia; 4 Present Address: Department of Gastroenterology, Center for Tumor- and Immune Biology, Philipps University of Marburg, 35043 Marburg, Germany

**Keywords:** Cancer drug resistance, Tumor microenvironment, Monocytes, HSF1, THP1

## Abstract

Macrophages constitute a major part of tumor microenvironment, and most of existing data demonstrate their ruling role in the development of anti-drug resistance of cancer cell. One of the most powerful protection system is based on heat shock proteins whose synthesis is triggered by activated Heat Shock Factor-1 (HSF1); the inhibition of the HSF1 with CL-43 sensitized A549 lung cancer cells to the anti-cancer effect of etoposide. Notably, analyzing A549 tumor xenografts in mice we observed nest-like pattern of co-localization of A549 cells demonstrating enhanced expression of HSF1 with macrophages, and decided to check whether the above arrangement has a functional value for both cell types. It was found that the incubation of A549 or DLD1 colon cancer cells with either human monocytes or THP1 monocyte-like cells activated HSF1 and increased resistance to etoposide. Importantly, the same effect was shown when primary cultures of colon tumors were incubated with THP1 cells or with human monocytes. To prove that HSF1 is implicated in enhanced resistance caused by monocytic cells, we generated an A549 cell subline devoid of HSF1 which did not respond to incubation with THP1 cells. The pharmacological inhibition of HSF1 with CL-43 also abolished the effect of THP1 cells on primary tumor cells, highlighting a new target of tumor-associated macrophages in a cell proteostasis mechanism.

**Supplementary Information:**

The online version contains supplementary material available at 10.1007/s00262-023-03612-2.

## Introduction

Despite the progress in molecular oncology, the effectiveness of tumor chemotherapy remains insufficient. A partial explanation of such insufficiency lies in the ability of tumors to adapt to changes in the environment, the so-called plasticity of cancer cells. Among others, heat shock response (HSR) is an important protective mechanism, which allows cells to adapt in constantly changing environmental conditions and respond to stressors that disrupt proteostasis [1]. The HSR program is constitutively activated in many types of tumor cells because they are subjected to proteotoxic stressors, hypoxia and oxidative or reductive stress, as well as to antitumor drugs, during the patient’s lifetime [2]. This leads to activation of the HSF1 transcription factor and the increased synthesis of its client proteins, molecular chaperones (HSP70, HSP90, HSP40, etc.), which allows cancer cells to avoid death and continue growing.

Over the past decade, it has become clear that HSF1 functions in tumors beyond HSR induction. In 2012, Mendillo et al. showed that HSF1 is able to control a transcriptional program distinct from HSR and promotes cell malignancy, not only by activating the synthesis of HSPs [3]. Furthermore, it has recently been shown that HSF1 can indirectly influence the resistance of colon cancer cells to oxaliplatin through the activation of protective autophagy and control the expression of multidrug resistance genes [4, 5]. Recently HSF1 was shown to form complex with c-MYC increasing its transcriptional activity in pro-tumor fashion [6], and therefore, HSF1 targeting is a promising approach in oncology. Of note, a few of specific HSF1 inhibitors have been launched within last few years and some of them show their efficacy in advanced in vivo models of cancer [7–9]. Earlier we reported another powerful inhibitor of HSF1, CL-43, which showed its anti-cancer activity being alone and in combination with clinically approved medicines [10].

One of the critical factors of tumor progression is tumor microenvironment (TME), which is composed of stromal cells and found to control the development of drug resistance [11]. Tumor-associated macrophages (TAMs) make up a significant part of the TME, which regulates tumor growth, angiogenesis, metastasis and drug resistance [12]. TAMs are a mixed population of pro- and anti-inflammatory activated macrophages and recently infiltrated macrophages and monocytes that migrate from surrounding tissues or through blood vessel walls and undergo further changes in the tumor microenvironment [13]. Several studies have shown that macrophages are also responsible for tumor therapeutic resistance based on cross-interaction between tumor cells and other cellular components of the tumor microenvironment, especially immune cells [3, 14]. TAMs were found to directly interact with tumor cells or exploit exosome-mediated mechanisms to promote the angiogenesis and metastasis of tumors in numerous animal models and in oncological patients [15, 16].

In our previous studies, we found that co-culturing A549 lung cancer cells with naïve monocyte-like cells resulted in enhanced tumor cell resistance to etoposide [17]. The present study aimed to understand how A549 cells and cells from patients with colorectal cancer acquire monocyte/macrophage-mediated resistance and whether it is related to cellular proteostasis machinery presumably driven by HSF1.

## Materials and methods

### Cells

Human leukemia monocytic cell line THP1, human lung adenocarcinoma cell line A549 and human colorectal adenocarcinoma cell line DLD1 were obtained from the Russian Collection of Cell Cultures (Institute of Cytology of Russian Academy of Science, St. Petersburg, Russia). The HeLa_luc_ cells containing the luciferase gene under the control of the HSE promoter [18] were kindly provided by Professor R.I. Morimoto, Northwestern University, USA. Colon carcinoma cells HCC6, HCC7 and HCC9 were obtained from tumor biopsies from patients of the St. Petersburg State University N.I. Pirogov Clinic of HighMedical Technologies in accordance with the rules of the ethics commission. Cell lines were named according to their etiology (Human Colon Carcinoma, HCC) and patient number. HCC9 cells were obtained from biopsy material from a 56-year-old female patient with a diagnosis of colon cancer of the hepatic bend pT3N2b(7/19)M0, G2, PNI-, VI-, IIIC stage; HCC6 cells were obtained from a 69-year-old female patient with a diagnosis of ascending colon cancer pT3N1a(1/26)M0, G3, VI-, PNI-, IIIB stage; and HCC7 cells came from a 69-year-old female patient with a diagnosis of sigmoid colon cancer (adenocarcinoma) pT3N2b(16/23)M0 G2 PNI + LV + IIIC stage [19]. According to the degree of malignancy, based on the international classification TxNxMx, the resulting cell lines line up in the following order: HCC6 < HCC9 < HCC7. On the day that the biopsies were performed, the tumor tissue was mechanically disintegrated and the cell suspension was seeded into wells of a 12-well plate (TPP, Trasadingen, Switzerland). Cells had three passages and were then frozen in liquid nitrogen. For the experiments, the cells were thawed and multiplied during at least two passages.

HCC cells, A549, DLD1 and HeLa_luc_ cells were grown in Dulbecco’s Modified Eagle Medium (BioloT, St.Petersburg, Russia) supplemented with 100 U/mL penicillin and 0.1 mg/mL streptomycin (Capricorn Scientific, Germany), as well as 10% fetal bovine serum (HyClone, Logan, UT, USA) at 37 °C and 5% CO_2_. HCT15 and THP1 were cultivated on RPMI 1640 medium (BioloT, St.Petersburg, Russia) supplemented with 100 u/mL penicillin, 0.1 mg/mL streptomycin and 10% FBS under the same conditions.

### Peripheral blood mononuclear cells isolation

Blood samples were collected in the morning, on an empty stomach, from a healthy 24 year-old male volunteer and monocyte isolation was performed with the use of Histopaque-1077 (Sigma-Aldrich, St. Louis, MO, USA), according to the manufacturer’s instructions.

### Plasmids

Transfer plasmid pGFP-C-shLenti for the knockdown of Hsp70 and its master regulator HSF1 was purchased from OriGene Technologies (Rockville, MD, United States): clones TRCN0000008513 (shRNA against HSPA1A (Hsp70)); a sequence encoding the mature sense sequence TTGATGCTCTTTGTTCAGGTCG, TRCN0000280463, or TRCN0000007481 (shRNA against Hsf1); and a sequence encoding the mature sense sequence CAAACGTGGAAGCTGTTCC or ATACTTGGGCATGGAATGTGC, respectively. The packaging plasmid pMD2.G and the viral envelope plasmid psPAX were purchased from Addgene (Watertown, MA, United States). A549_luc_ cells were also transduced with the luciferase gene using the pHIV-iRFP720-E2A-Luc, as described previously [20].

### Drugs

CL-43 was obtained from the InterBioScreen collection (Chernogolovka, Russia), dissolved in dimethyl sulfoxide (DMSO) an initial concentration of 20 mM and stored at − 20 °C until use. Etoposide and chloroquine (both from Sigma-Aldrich, St. Louis, MO, USA) were also used in this study.

### Scheme of co-cultivation

Twenty hours before the start of co-cultivation, THP1 cells or monocytes were transferred to the DMEM medium. When the cancer cells were attached to the dish surface, the monocyte and THP1 cells were added at a ratio of 1 monocyte per 100 cancer cells. Cells were co-cultivated for 20 h.

### Luciferase assay

HeLa-luc cells containing the luciferase gene under the control of the heat shock protein gene promoter, HSE, were plated on 24-well plates at a concentration of 15 × 10^4^ cells/ml and co-cultured with THP1 or monocytes in the indicated ratios for 20 h. Cells incubated with 5 µM U133 were used as a positive control. The luciferase reporter test was analyzed using the Bright Glo kit (Promega, Madison, WI, USA) according to the manufacturer's protocol. Luciferase activity was detected using Varioskan equipment (Thermo Fisher, Waltham, MA, USA).

### Confocal microscopy

To visualize the localization of the phosphorylated form of HSF1 in cells, HCC cells were plated onto 24-well plates with pre-loaded coverslips. When attached, the cancer cells were incubated with THP1 cells in ratio 100:1 for 20 h and then THP1 cells were washed out, fixed in 4% paraformaldehyde, permeabilized with 0.1% Triton and incubated with primary antibodies to pHSF1 Ser326 (SU31-03, ThermoFisher, Waltham, MA, USA) followed by secondary antibodies conjugated with Alexa Fluor 647 (Thermo Fisher, Waltham, MA,USA). Cell nuclei were stained with 4’,6-diamidino-2-phenolindole (DAPI) fluorescent dye. Coverslips with cells were placed in a mounting medium Fluoroshield (Sigma-Aldrich, St. Louis, MO, USA). Fluorescence images were captured by an Olympus FV3000 confocal microscope and analyzed with cellSens software.

### Cytotoxicity assay

The viability of cancer cells was evaluated with aid of the xCELLigence Real-Time Cell Analyzer DC equipment (Hoffmann-La Roche, Switzerland). HCC cells were seeded in 16-well E-plates (Agilent Technologies, San Diego, CA, USA) at a concentration of 8.0 × 10^3^ cells/mL; 24 h later, 10^3^ THP1 was added for co-cultivation. To avoid the influence of THP1 on cell index, cells were separated by 0.4 μm pore size inserts (Agilent Technologies, Inc. Headquarters, USA). On the following day, cells were treated with 200 μm chloroquine alone or in combination with 500 nM CL-43. The dynamics of cell proliferative activity were recorded over the next 48 h using the xCELLigence equipment and analyzed using RTCA Analysis Software (Agilent Technologies, San Diego, CA, USA).

### Spheroids viability assay

For spheroid formation, A549 cells were seeded into an agarose-coated dish at a cell density of approximately 8000 cells/well. Into experimental wells, we also added THP1 cells at a ratio of 1 THP1 cell per 100 cancer cells. After 5 days, 200 μm of chloroquine was added. Viability was measured by the CellTiter-Glo® 3D Cell Viability Assay (Promega, Madison, WI, USA) according to the manufacturer's protocol.

### Detection of apoptosis

Control cells and cells co-cultivated with THP1 or monocytes treated with etoposide or chloroquine were used in the flow cytometry assay. Cells were seeded into 12-well plates at a concentration of 10^5^ cells/well. The next day, simultaneously with the medium changing, 5 × 10^3^ THP1 cells were added. After 20 h of co-cultivation, chloroquine (200 μm) or etoposide (25 μm) were added without changing the medium. Further plates were cultivated for the next 36 h in the incubator and then cells were prepared for flow cytometry as described in our previous works [21] The detection of apoptosis was carried out with the aid of Annexin V Alexa 647 (Life Technology, Carlsbad, CA, USA) combined with Propidium Iodide staining. All procedures were performed according to the manufacturer's protocol.

### Western-blotting

Cancer cell cultures were incubated in medium containing THP1 for 20 h, before being washed and lysed in High RIPA buffer (20 mM Tris–HCl pH 7.5, 150 mM NaCl, 0.1% Triton X-100, 0.5% SDS, 1 mM PMSF, 2 mM EDTA, 0.5% DOC). After sonication, lysates were centrifuged at 13,400 rpm. The protein concentration in the supernatant was measured using the Bradford method and 30 µg of total protein was precipitated with acetone at − 20 °C for the night and centrifuged at 12,000 × g; the pellet was dissolved in Laemmli buffer (100 mM Tris–HCl pH 6.8, 4% SDS, 20% glycerol, 0.005% PBS, 50 mM DTT). After electrophoretic separation, proteins were transferred to PVDF Blotting Membrane (A29562258, Amersham Hybond, Germany) using the Mini Trans-Blot cell (Bio-Rad, Hercules, CA, US). The membrane was blocked with phosphate-buffered saline (PBS) containing 3% (w/v) skimmed milk and incubated, with primary antibodies against pHSF1 Ser326 (SU31-03, ThermoFisher, USA) and HSP70 (clone 3B5) followed by secondary antibodies against rabbit and mouse immunoglobulins conjugated with horseradish peroxidase (Abcam, Cambridge, UK). Anti-alpha-tubulin antibodies (MA1-80017, ThermoFisher, Waltham, MA, USA) were used as the sample loading control. Images were captured by a ChemiDoc (Bio-Rad, Hercules, CA, US) detection system with Femto ECL reagent (catalogue number 34096, Thermo Fisher, Waltham, MA, USA).

### Animal experiments

All in vivo experimental protocols were approved by the licensing committee of the Institute of Cytology of the Russian Academy of Sciences (Identification number F18-00380). All methods were carried out in accordance with the relevant guidelines and regulations. All methods are reported in accordance with the ARRIVE guidelines.

Animals were purchased from the National Research Lobachevsky State University of Nizhny Novgorod (Russia). Twenty four BALB/C nude male mice were subcutaneously injected with 10^6^ A549_luc_ cells in Matrigel (Corning Incorporated, New York, USA).

From day 10 after tumor cell inoculation, when the tumors could be detected by palpation, the mice were divided for four groups, with 6 animals in each. The first group had no treatment (‘Contr’), the second group (‘Eto’) was treated with 5 mg/kg Etoposide, the third group (‘CL-43’) received the 2 mg/kg CL-43 therapy and the last group (‘Comb’) was treated with a combination of these two drugs. Drug injections were performed twice a week during the experiment.

At the end of the treatment, the tumor volume was measured using the IVIS Spectrum in vivo Imaging System (PerkinElmer, Waltham, MA, USA). Luciferin (Thermo Scientific, Waltham, MA, USA) at a concentration of 30 mg/ml was diluted in DPBS (Paneko,, Moscow, Russia). Mice were injected with 100 µl of luciferin and placed in a chamber containing inhalation anesthesia with Aerrane (Baxter Healthcare Corporation, Deerfield, IL, USA). Luminescence detection was carried out in the mode of automatic selection of the signal accumulation time.

After the analysis, half of the mice from each group were perfused with 4% paraformaldehyde and tumors were used for immunohistochemistry. Tumors from the other half of the animals were used for RNA isolation and real-time PCR.

### RNA isolation and real-time PCR

RNA was isolated using Extract RNA (Evrogen JSC, Moscow, Russia) and reverse transcription was performed using the MMLV RT kit (Evrogen JSC, Moscow, Russia) according to the manufacturer's instructions. All RT-PCR studies were carried out using a CFX96 Real-Time PCR detection system (Bio-Rad, Hercules, CA, USA) and qPCRmix-HS SYBR (Evrogen, Moscow, Russia) according to the manufacturer’s protocol. Amplicon authenticity was confirmed by melt curve analysis. All primers were obtained from Evrogen JSC (Moscow, Russia). β-Actin was used as a loading control in this study. The data were analyzed for fold-change using Bio-Rad CFX software (version 3.1; Hercules, CA, USA).

The sequences of the primers used in this study are as follows:

- β-actin Forward: CCATCATGAAGTGTGACGTGG
- β-actin Reverse: GTCCGCCTAGAAGCATTTGCG
- HSPA1A Forward: GCCGAGAAGGACGAGTTTGA
- HSPA1A Reverse: TCCGCTGATGATGGGGTTAC
- Ki67 Forward: TCCTAGGAAAACTCCAGTTGCC
- Ki67 Reverse: AGACACTCTCTTTGAAGGCAGG

### Immunohistochemistry

Serial 10 μm frozen slices from tumors of each experimental group were prepared using the OTF6000 Cryostat (Bright Instruments, UK). Slices were probed with primary antibodies to pHSF1 S326 (SU31-03, ThermoFisher, Hercules, CA, USAm USA) and antibodies to macrophage marker Iba1 (FUJIFILM Wako Pure Chemical Corporation, Richmond, VA, USA). After careful washing, probes were further incubated in secondary antibodies conjugated with Alexa Fluor 647 fluorochrome (Thermo Fisher, Hercules, CA, USA, USA). After washing from antibodies, cell nuclei were stained with DAPI fluorescent dye. An Olympus FV3000 confocal laser microscope was used for visualization.

## Results

### CL-43 increases the sensitivity of lung adenocarcinoma A549 to etoposide

**Fig. 1:**
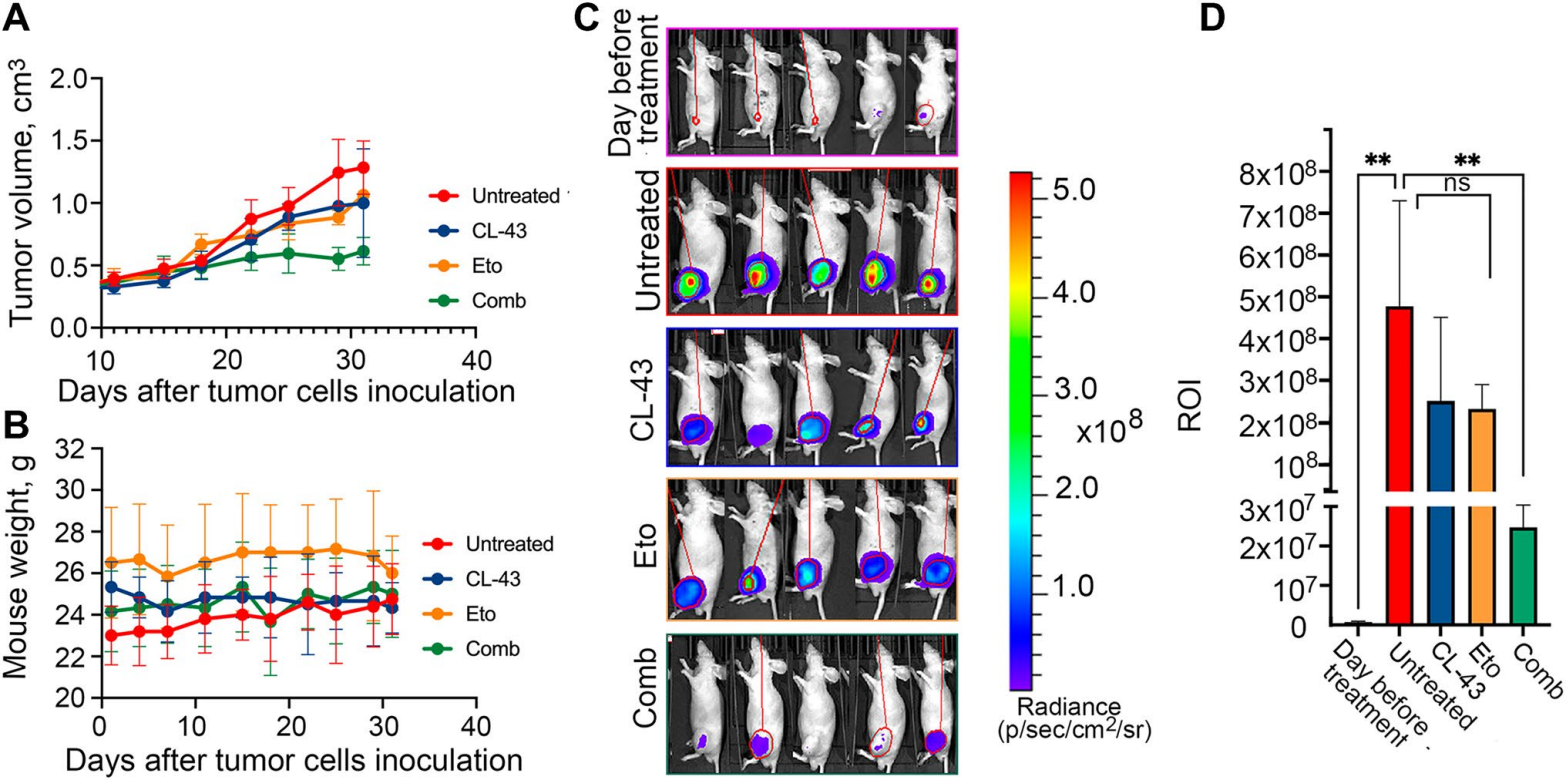
CL-43 increased the sensitivity of A549 cells to etoposide in vivo. **A** Balb/c nude mice were injected with 10^6^ A549_luc_ cells and were divided for four groups starting from day 10 after injection, when the tumors became palpable: ‘Contr’, ‘Eto’, ‘Cl-43’ and ‘Comb’(n = 6 in each group) and treatment was started. Tumor volume was measured twice a week; **B** Mice were weighed on Day “0” and then continued to be weighed twice a week until the end of the experiment. **C** A549luc tumor growth was estimated using IVIS Spectrum imaging system on day 32; **D** Luminescence count of tumor lesions. ** p < 0.001

Previously, in the process of high throughput screening, we discovered a pan-activator inhibitor of heat shock proteins, cardenolide CL-43, and demonstrated that the compound effectively increased the sensitivity of different tumor cells to first-line therapies [10]. To demonstrate that CL-43 is also effective in vivo, we used a heterotopic xenograft mouse model. We compared the tumor growth rate of A549_luc_ cells in 4 groups of mice: ‘Contr’ (untreated) mice, mice injected intraperitoneally with CL-43 (‘CL-43’), mice treated with etoposide alone (‘Eto’) and mice treated with a combination of CL-43 and etoposide (‘Comb’). Ten days after tumor cells were injected, when the tumors were already pulpable, the therapy was started and lasted for the next 32 days. We found that tumor growth in the ‘Comb’ group was delayed and tumor size did not change significantly from day 22 of therapy. In the ‘Eto’ and ‘Cl-43’ groups, tumor growth continued throughout the entire therapy, although it was slower compared to than in the ‘Contr’ group (Fig. 1A). Notably, all mice tolerated the therapy and their weight remained unchanged throughout the therapy (Fig. 1B). IVIS bioimaging (Fig. 1C) performed on day 42 after tumor cell inoculation demonstrated that only mice from the ‘Comb’ group showed a significant decrease in bioluminescence (ROI) (Fig. 1D).

**Fig. 2:**
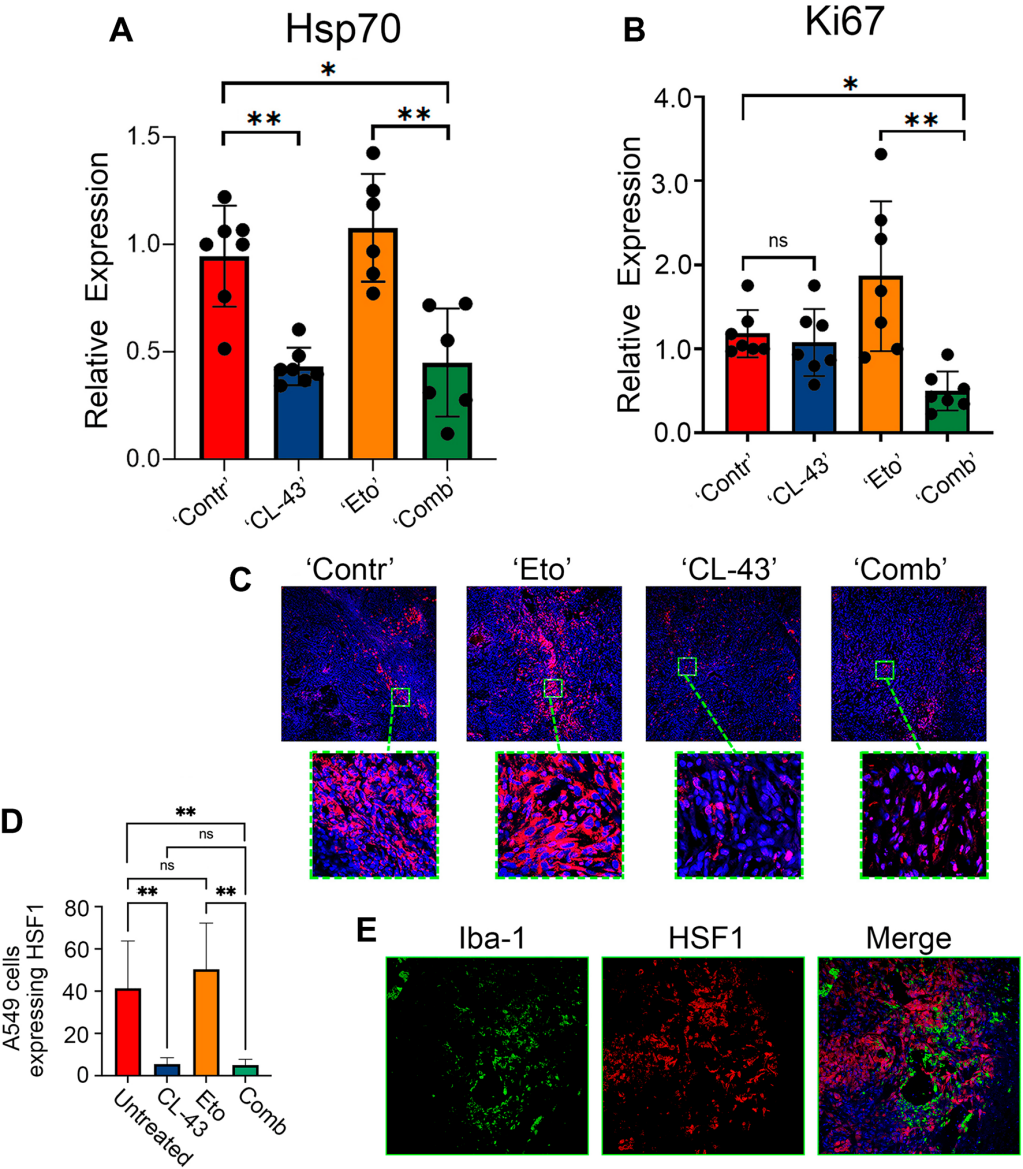
Tumor locales with high HSF1 expression were inhabited by macrophages. **A**, **B** Normalized relative expression of Hsp70 (**A**) and Ki67 (**B**) in tumors from mice after 32 days of treatment; *p < 0.05; **p < 0.001. **C** Representative immunostaining fluorescence images of A549 tumor slices from four experimental groups with inserts. pHSF1^Ser326^ was stained in red, the nucleus was stained with DAPI in blue. **D** Counting of A549 cells expressing HSF1 in tumor slices from four experimental groups. Tumor slices from three mice from each group were counted. Data are presented as a percentage of all tumor cells. **E** Slices from the ‘Contr’ group were stained with anti- pHSF1^Ser326^ antibody (red) and with an antibody to macrophage marker Iba1 (green); the nucleus was stained with DAPI (blue)

When the experiment ended, tumors were isolated and part of the tumors from each group was analyzed by RT-PCR using primers for the HSP70 and cell proliferation markers Ki67 (Fig. 2A, B). Another part was used for immunohistochemical analysis. Analysis of Hsp70 and Ki-67 mRNA expression showed that Hsp70 was decreased 2.4-fold in tumors from the ‘CL-43’ and ‘Comb’ groups compared to tumors from the ‘Contr’ group. Hsp70 levels in tumors from the ‘Eto’ group were not statistically different from ‘Contr’ (Fig. 2A). The analysis of Ki-67 showed that the ‘Comb’ group had the lowest value compared to the other groups, indicating a reduced tumor growth rate. Interestingly, Ki-67 was significantly elevated in the ‘Eto’ group, which may indicate that a part of the tumor cell population dies as a result of the therapy, while the remaining part continues to grow at a doubled rate (Fig. 2B).

Histochemical analysis of tumor slices with use of anti-HSF1^Ser326^ antibodies showed (1) that tumors from the ‘CL-43’ or ‘Comb’ groups had significantly lower HSF1 levels, as expected (Fig. 2C), and (2) cells with high HSF1^Ser326^ levels were not distributed around the whole tumor volume but formed islets in the ‘Contr’ and ‘Eto’ groups (Fig. 2C). This reminded us of the distribution pattern of tumor-associated macrophages (TAM) in B16 tumors [20]; we stained the tumor slides from untreated animals with Iba1 antibody (recognizing all macrophages) and demonstrated that areas with elevated HSF1 were also inhabited by Iba1 positive cells.

### Co-cultivation of THP1 and various tumor cells leads to activation of HSF1

**Fig. 3:**
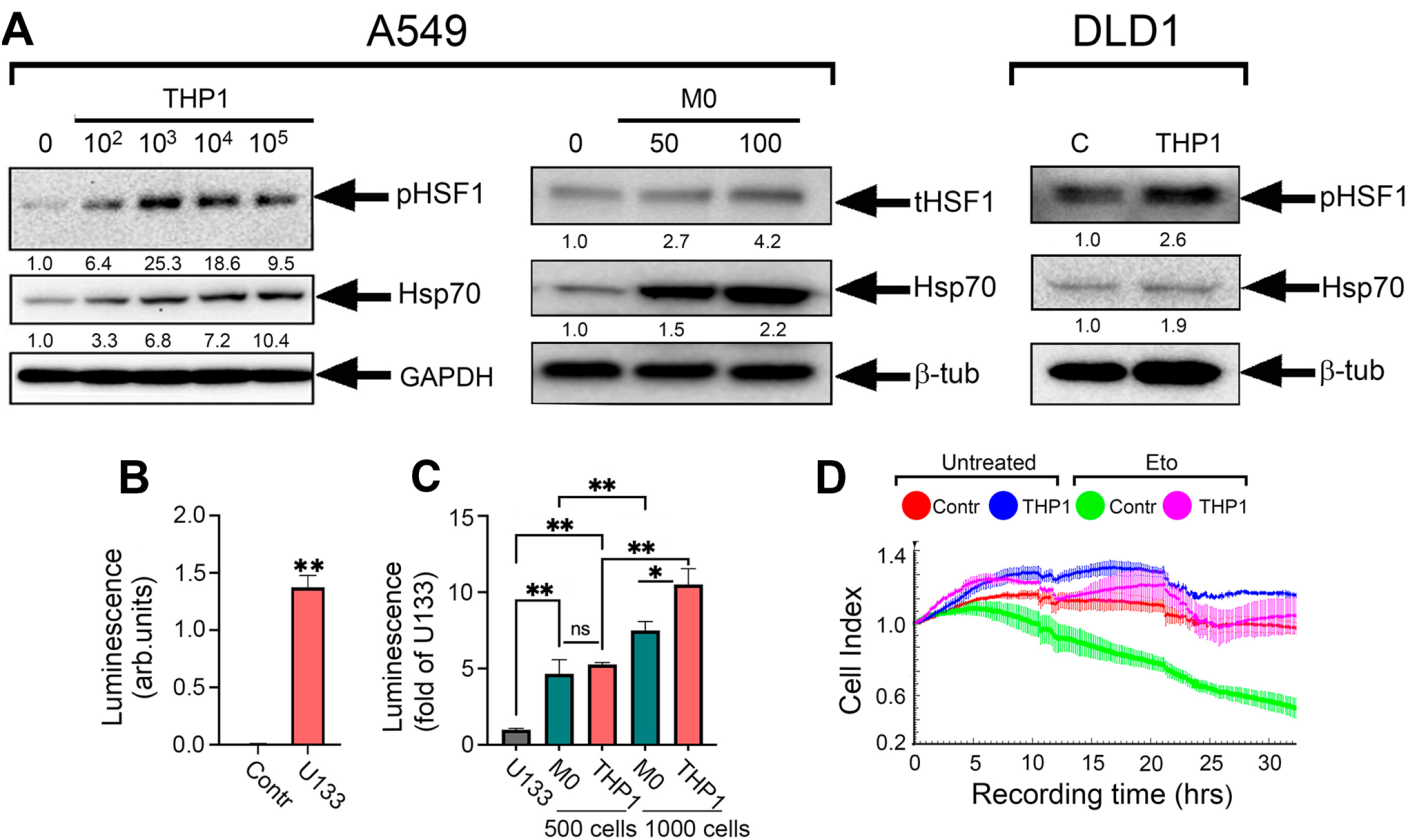
Co-cultivation of tumor cells with human monocytes or monocyte-like THP1 cells led to an increase in HSF1 activity in tumor cells. **A** Western blot analysis of A549 and DLD1 cells after incubation either with THP1 cells of with human monocytes for 20 h performed with pHSF1^Ser326^ and HSP70 antibodies. β-tubulin was used as the loading control. **B** HSE-luciferase reporter assay performed with HeLa_luc_ cells incubated with 5 µM U133, a well-known activator of HSF1, 20 h after the addition of U133. Data are presented in arbitrary luminescence units; **C** HSE-luciferase reporter assay of cells after co-cultivation with THP1 or human mononcytes. Cells were analyzed 20 h after the addition of THP1 or human monocytes. **D** A549 cells were seeded on E-plate and proliferation activity was measured using xCELLigence. THP1 cells were added after 20 h onto special inserts which contains a 0.4 μm pore size membrane. Etoposide was added 20 h after the addition of THP1

To understand whether cells overexpressing HSF1 can be targeted by macrophages in tumors or whether contact with macrophages induces HSF1 expression, we first co-cultivated tumor cells with either human monocytes or with monocyte-like THP1 cells that are widely used in studies on macrophage-M2 transition [22, 23]. We used A549 and DLD1 cells co-cultured with THP1 cells or with human monocytes at a 40:1 ratio. After 20 h of co-cultivation, monocyte-like cells were removed and A549 and DLD1 cells were employed for immunoblotting. The co-culture of A549 or DLD1 cells with monocytes or monocyte-like THP1 increased pHSF1(Ser^326^) or total HSF1 in tumor cells (Fig. 3A).

To be completely sure that THP1 cells are available to substitute human monocytes in the in vitro studies, we used the HSE-luciferase reporter system on a base of HeLa cells (HeLa_luc_) [18]. HeLa_luc_ cells (8000 cells in well) expressing the genetic construct consisting luciferase gene under heat shock promoter (HSE) were co-cultured with 500 or 1000 THP1 cells or with monocytes isolated from human blood for 20 h. As a positive control, we used a derivative of echinochrome, U133, a compound that is known to be a strong inducer of HSF1 [24]. Importantly, the luminescence caused by incubation with monocytes or with THP1 cells was much higher than that caused by U133. The HSF1 activation increased 13-fold when U133 was used compared with untreated HeLa_luc_ cells (Fig. 3B); however, co-cultivation with 500 monocytes caused 4.5-fold more HSF1 activation and 500 THP1 cells was 5.37-fold higher compared with U133 (Fig. 3C). An increase in the number of monocytes or THP1 led to an even greater increase in luminescence: 1000 monocytes increased the luminescence 7.7-fold and THP1 cells by up to 10.5-fold (Fig. 3C). Using the xCELLigence technique, we confirmed that the resistance of A549 cells to the antitumor drug increased after incubation with monocyte-like cells, which we attribute to the HSF1 activation (Fig. 3D).

**Fig. 4:**
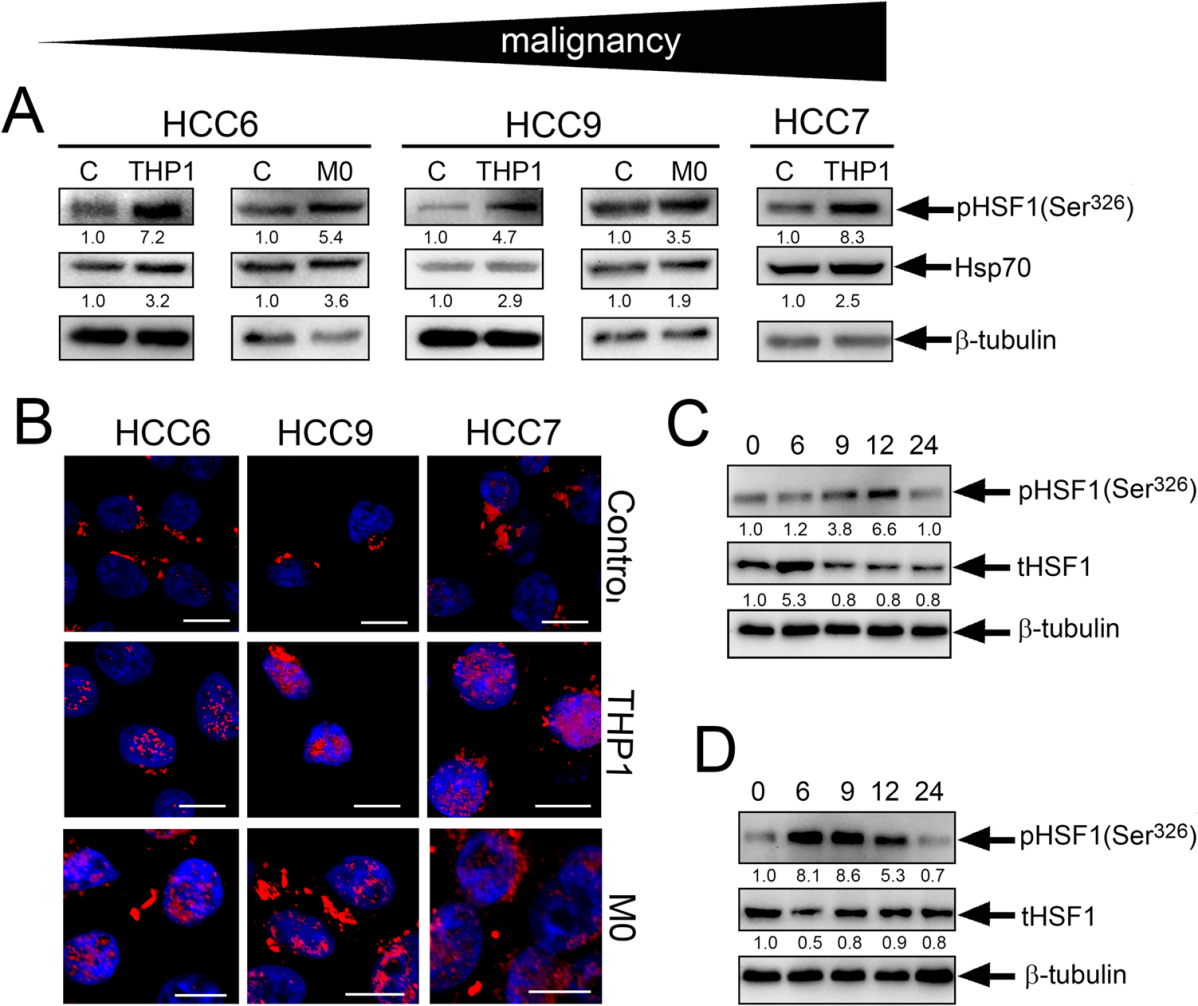
Pre-incubation of patient HCC cells with monocyte-like cells also leads to HSF1 activation. **A** Western blot analysis of HCC cells with anti-pHSF1^Ser326^ and anti-Hsp70 antibody after co-cultivation with THP1 or human monocytes for 20 h. β-tubulin was used as the loading control. The upper arrow demonstrates the level of malignancy of HCC cells according to medical diagnosis. **B** HCC6, HCC9 and HCC7 cells were seeded to cover glasses and then were incubated with THP1 or monocyte cells. Cells were stained with anti-pHSF1^Ser326^ antibody (red) and with DAPI (blue). Scale bar 5 µm. **C** The dynamics of HSF1 activation due to THP1 cells using the HCC6 cell as an example. HCC6 cells were co-cultured with THP1 at the indicated time intervals. **D** The dynamics of HSF1 expression after removal of THP1 from the co-culture at 20 h

To test whether HSF1 activity elevation in the presence of monocyte-like cells could also exist in cells freshly isolated from cancer patients, we used colorectal carcinoma cells (HCC cells) isolated from untreated patients. We incubated HCC cells with THP1cells or with naïve human monocytes for 20 h and then subjected them to immunoblotting with antibodies against the active form of pHSF1^Ser326^. We found that, like as in established cell lines, in freshly obtained cells, monocyte-like cells induce activation HSF1^S326^ (Fig. 4A). Immunocytochemical staining of HCC6, HCC7 and HCC9 cells co-cultivated 20 h with THP1 revealed pHSF1^Ser326^ in the nucleus, which is essential for its function as a transcription factor and further indicates that it is in the active state (Fig. 4B).

We then investigated the dynamics of HSF1 activation due to THP1 cells using the HCC6 cell as an example. HCC6 cells were co-cultured with THP1 at the indicated time intervals (Fig. 4C) and subjected to western blotting. The data showed that the maximum activation of pHSF1^Ser326^ was detected 24 h after the co-culture of HCC6 and THP1 and was 3.6-fold higher than the control level. We also demonstrated that after removal of THP1 from the co-culture at 20 h, HSF1 activity gradually decreased and reached the control level at 24 h (Fig. 4D) 

### THP1 contributes to the resistance of tumor cells from patients with colorectal cancer to chloroquine

**Fig. 5:**
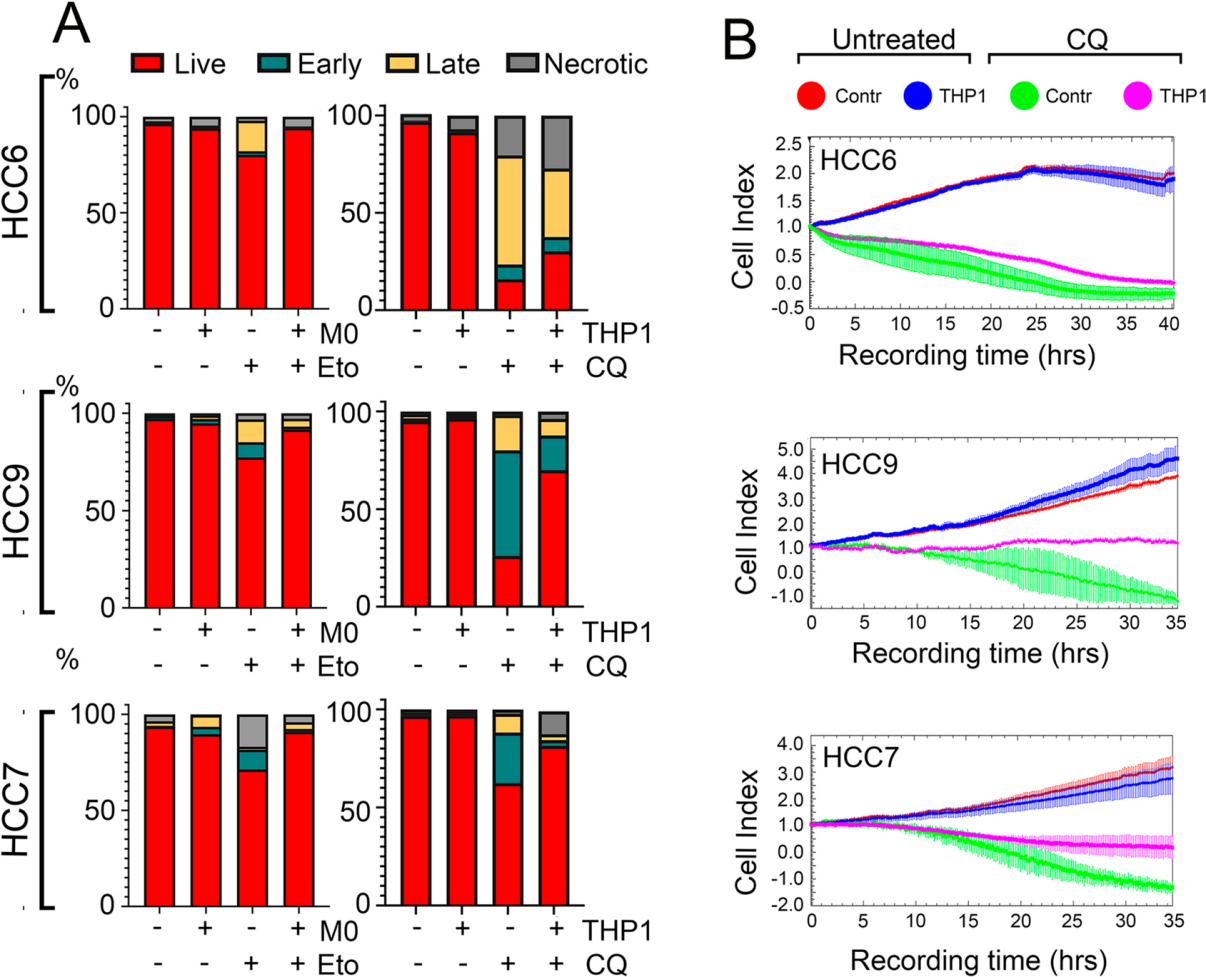
Monocytes reduce the sensitivity of HCC cells to cytotoxic agents. **A** HCC cells were preincubated with human monocytes for 20 h and then either etoposide was added for 36 h (left panel) or co-cultivated with THP1 after 20 h. After that, CQ was administered for 24 h (right panel). The apoptosis was measured with the aid of flow cytometry with use of Annexin V Alexa 647 and propidium iodide (PI). Each panel shows representative data from one of three independent experiments. **B** HCC cells were treated as described above. The cell index was measured using the xCELLigence system for 35–40 h

To understand whether THP1 or human monocytes can affect tumor cell resistance, same as shown for A549 cells, we first detected apoptosis in HCCs cells treated with etoposide with aid of Annexin V. HCCs cells were first incubated with monocytes in ratio 50:1 for 20 h and then 30 µM etoposide was administrated for next 24 h. Etoposide caused cell death, both apoptotic and necrotic, in the range from 11–30%, but pre-incubation with monocytes resulted in a level comparable to that in untreated HCCs cells (Fig. 5A left panel). In our previous work, we showed that tumor cells derived from colorectal cancer patients were highly resistant to first-line drugs (etoposide, cisplatin, 5FU) but sensitive to chloroquine (CQ) [5], a well-known antimalaria drug that is now being considered as an anticancer drug and is undergoing clinical trials [25], so we used CQ in the further experiments.

HCC cells were preincubated with THP1 cells in ratio 100:1 for 20 h and then 150 µM CQ was added for the next 36 h. Pre-incubation with THP1 cells increased the resistance of HCC cells to CQ: after CQ treatment in HCC6 cell populations, 15.9% cells left alive but pretreatment with THP1 cells increased living cell numbers to 30.3%; in HCC9 after incubation with CQ, only 26.05% living cells remained, whereas pre-incubation with THP1 cells left 69.9% of living cells; in HCC7 cells after CQ treatment, 62.4% of living cells were left and THP1 pre-incubation increased viability to 81.5% (Fig. 5A, right panel). The data received with the aid of the xCELLigence technique, which allows real-time cell proliferation assessment, confirmed the data of flow cytometry and demonstrated that the cell index of cells treated with CQ was 0.5 lower than that of cells pre-incubated with THP1 (Fig. 5B), indicating that co-culture with monocytes delays cell death caused by CQ treatment in the three patient lines.

### HSF1 down-regulation decreases the resistance of A549 cells to etoposide treatment

**Fig. 6:**
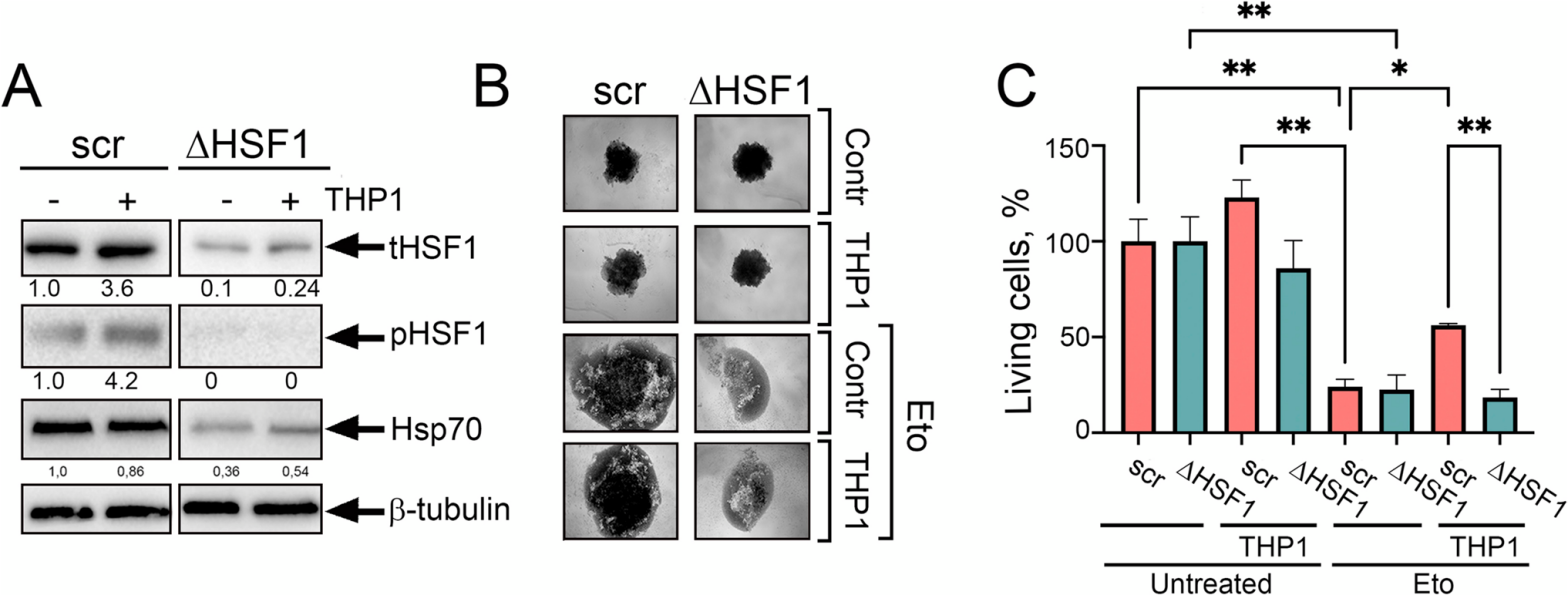
In cells with HSF1 knock-down and co-incubation with monocyte-like cells, resistance to etoposide does not form. **A** Western blot analysis of the level of total HSF1 (tHSF1) and active pHSF1^Ser326^ in A549_scr_ ( A549 cells, transfected with empty plasmid) or A549_kdHSF1_ (∆HSF1) cells after co-cultivation with THP1for 20 h. β-tubulin was used as the loading control. **B** Spheroids were formed from A549scr or A549_kdHSF1_ cells with or without incubation with THP1 cells. After 6 days, etoposide was added at a concentration of 200 μm. Images were taken at 60 h. **C** Spheroids were treated as described in (**B**) and viability analyzed using CellTiter-Glo 3D kit (Promega, US). *p < 0.05; **p < 0.001

We hypothesized that cells used with knocked-down HSF1 made them less resistant to anticancer drugs being incubated with monocyte-like cells. We obtained A549_kdHSF1_ cells using specific shRNA [26] and incubated them with THP1 in ratio 200:1; twenty hrs later, A549_wt_ and A549_kdHSF1_ cells were employed for western blotting. As demonstrated previously, both total and phosphorylated HSF1 was increased ~ fourfold in A549_wt_ cells, whereas the total HSF1 level in A549_kdHSF1_ cells was tenfold less than in parental cells. Co-cultivation with THP1 led to an increase in total factor level of HSF1 but activated HSF1 was undetectable; despite this, the client Hsp70 level was elevated ~ 1,5 times (Fig. 6A).

To evaluate the sensitivity of cells with normal and down-regulated HSF1 levels, we formed spheroids using 8 × 10^3^ cells of each A549 subline with or without THP1. After 5 days, we used etoposide for 72 h. After taking micrographs (Fig. 6B), cell viability was assessed with the CellTiter-Glo assay. The reduction of living cell quantity was initiated by etoposide in both cell sublines; however, the pre-incubation of A549_wt_ with THP1 cells increased their resistance to therapy 2.5-fold. Notably, A549_kdHSF1_ cells did not demonstrate such THP1-dependent resistance (Fig. 6C), proving that the effect of monocytes to tumor cell resistance is indeed related to HSF1 activity.

### CL-43 can reverse the cytoprotective effect of THP1 on colorectal cancer cells

**Fig. 7:**
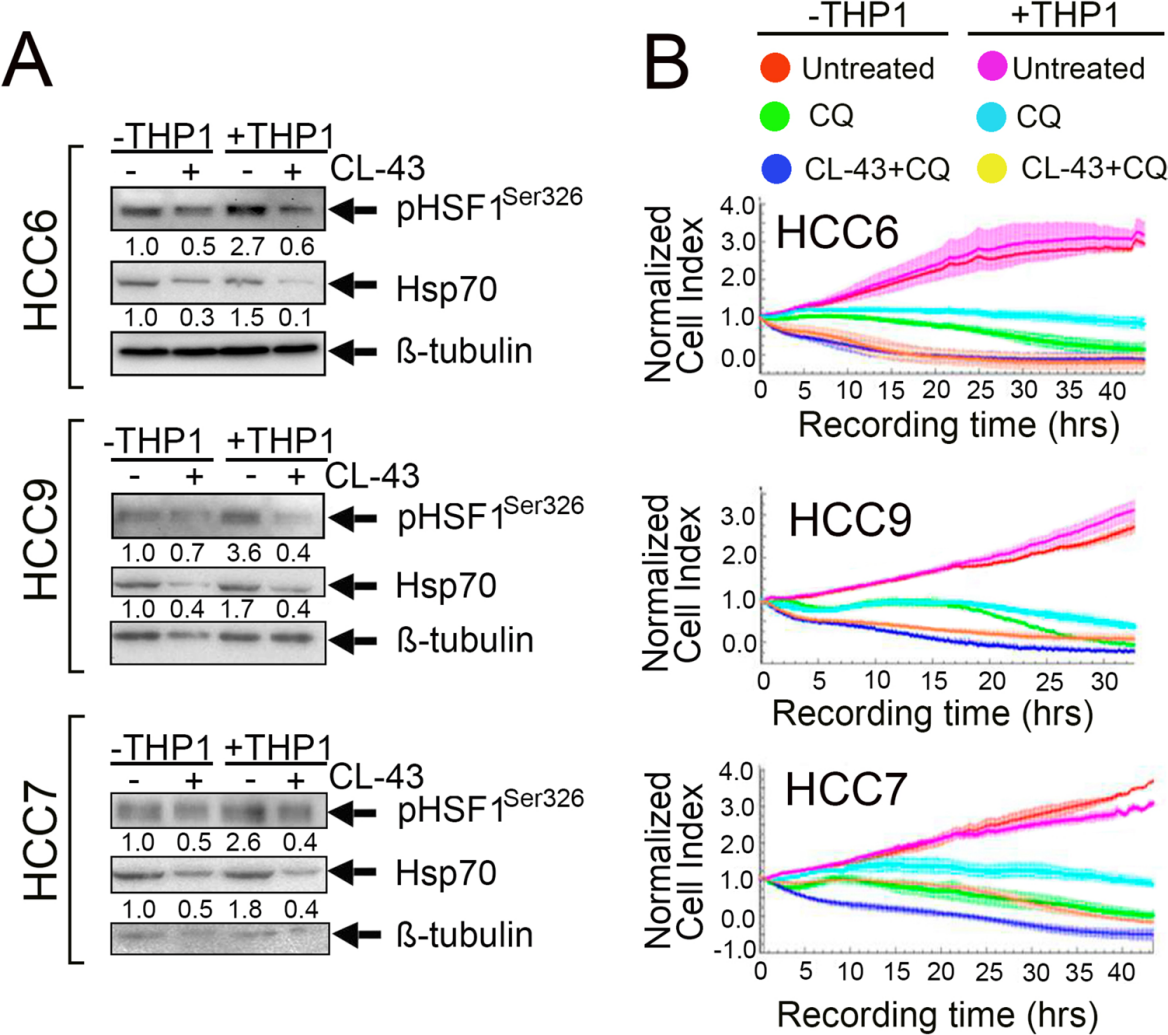
CL-43 reduced the cytoprotective effect caused by monocyte-like cells inhibiting HSF1 activity. **A** HCC cells were incubated with 500 nm CL-43, co-cultivated with THP1 and, 20 h later, subjected to western blotting with antibodies against HSF1^Ser326^ and Hsp70. Band intensity was measured with the aid of the ImageJ program. Numbers indicate a ratio between HSF1 or Hsp70 band intensity to band intensity of tubulin. **B** HCC cells were seeded on E-plates and treated with 500 nM CL-43 in combination with 75 μM CQ in the presence or absence of THP1. The signal was recorded using the xCELLigence system for 30–40 h

Since the effect of monocytes or THP1 cells on tumor cell resistance is related to HSF1, we tested CL-43 to lower the factor activity before treating HCC cells with monocytes. Immunoblotting data demonstrated that CL-43 application effectively reduced the amount of the active form of pHSF1^Ser326^ in HCC6, HCC7 and HCC9 cells. Hsp70 quantity was also decreased (Fig. 7A).

To prove that HSF1 inhibition may be helpful in overcoming monocyte-mediated drug resistance, we explored the effects of CL-43 in HCC cells incubated with THP1 and treated with CQ using the xCELLigence technique. We observed a protective effect of THP1 on CQ-treated cells (light blue vs green lines). However, the application of CL-43 combined with CQ after THP1 cells resulted in a reduction of the cell index to the level of cells treated with the combination in the absence of THP1 for HCC6 and to the level of cells treated with CQ alone in the absence of THP1 for HCC7. We also observed accelerated cell death in HCC9 cells after CL-43 treatment in combination with CQ (Fig. 7B).

## Discussion

During the last few years, attempts to increase the efficiency of anti-cancer therapy were aimed at the development of targeted molecules able to recognize proteins constituting basic systems of tumor cell homeostasis without adverse effects on normal tissue [27]. One such target is HSF1, heat shock transcription factor, which has been found to be highly expressed in many cancers and which is thought to trigger the expression of heat shock proteins, HSPs, which are well known for their cytoprotective activity and tens of polypeptides involved in cancer progression [9, 28, 29].

Tumor growth dynamics critically depend on its microenvironment, particularly the monocytic-macrophage component, constituting significant part of the total mass of solid tumors and which is an important determinant of tumor progression [11, 13]. Earlier, we found that the pretreatment of tumor cells with monocytes led to an increase in anti-cancer drug resistance and this result correlated well with the data of a few recent reports [30–33].

In the present study, we sought to explore whether enhanced resistance of A549 lung cancer cells to etoposide may be caused by monocytes, particularly by their activating effect on HSF1. First, we found that CL-43, known inhibitor of HSF1 activity [10], in combination with etoposide significantly reduced the growth of A549 xenografts in nude mice. Furthermore, histochemical analysis of tumors with antibodies to phosphorylated HSF1 demonstrated that the protein level was reduced in tumors from animals treated with CL-43 localized to cells forming a "nest" pattern, resembling that of tumor-associated macrophages (TAMs) in a previous study [17]. The double staining of cryosections of control tumors with antibodies against the active form of HSF1 and the macrophage marker Iba1 revealed their close association. This result prompted us to investigate the influence of macrophage/monocytes on the cell proteostasis system in more detail.

TAMs can mediate drug resistance, mainly suppressing the activity of CD8 + T lymphocytes and/or inducing resistance to anti-cancer drugs [34]. Chemoresistance in breast cancer was caused by pharmacological inhibitors of the Hedgehog pathway that increased IL-6 expression by macrophages [35]. Furthermore, in colorectal cancer, IL-6 produced by TAMs activates the STAT3 pathway and consequently blocks expression of the miR-204-5p tumor suppressor [36]. In the present study, we demonstrate for the first time that co-culturing THP1 cells or monocytes isolated from healthy donors with various tumor cell types, including patient-derived tumors, leads to an increase in phosphorylated (active) HSF1. As described above, HSF1 is overexpressed and/or activated in various cancers and may mediate resistance of cancer cells of different histogenesis to various types of anticancer therapies [37–40]. Bioinformatic analysis also revealed an association between HSF1 expression levels and macrophage infiltration rates in many clinical samples [41]. These data are also supported by the results of bioinformatic analysis, in which the authors found a positive correlation between the level of HSF1 expression in esophageal squamous cell carcinoma and the infiltration of M0 macrophages together with M2 macrophages and activated mast cells [42]. In addition, Liu et al. demonstrated that the interaction between TAM and hepatocellular carcinoma cells leads to HSF1-mediated changes in energy metabolism that contribute to tumor cell malignancy [43]. In the present study, we show that HSF1-activating effect and the reduction of cell sensitivity to anti-cancer drugs was independent of the type of monocytic cells or tumor cell line, suggesting that the phenomenon of acquired resistance is related to HSF1. To check the key role of the factor, we used spheroids made up of A549 cells devoid of HSF1 and found that they became insensitive to the treatment with THP1. Furthermore, the application of CL-43 also resulted in a reduction of active (phosphorylated) HSF1 form in colon cancer cells from human samples and the simultaneous increase of their sensitivity to cytotoxic effect of CQ even after the reaction with THP1 monocytes. Of note, in experiments on cell interactions a number of cancer cells exceeded that of monocytes or THP1 cells 40-fold, which is a realistic value for cancer stroma. In very recent work, Hao et al. used a ratio cancer cells: macrophage 30:1, and demonstrated that co‐culturing with macrophages greatly increased triple-negative breast cancer cell migration, tumor growth, and cancer metastasis [44]. This led us to suggest that such a system of tumor engagement by macrophages should function even in the beginning of tumor growth.

Almost a decade ago, Dr. Lindquist’s group reported that HSF1 plays a dominant role in reprogramming cancer-associated fibroblasts to support the malignancy of lung cancer by promoting the production of two key stromal signaling molecules, TGF-β and SDF1 [45]. The data presented here can accomplish the hypothetical picture of events occurring in the tumor according to which monocytes/macrophages and other stromal cells, primarily fibroblasts and neutrophils, undergo rewiring depending on their amount and powerfulness of cancer cells; the balance between these abilities may be established by proteostasis mechanisms, including molecular chaperones governed by HSF1 and autophagy. This process may involve cancer cells even in the very beginning of tumor growth and dissemination, e.g., tumor-initiating cells, stem-like cells and drug-tolerant persisters [46, 47].

We also showed here that the negative effects of increased HSF1 activation in tumor cells, observed after their co-culture with monocyte-like cells, can be neutralized by the HSF1 activity inhibitor CL-43.

### Supplementary Information

Below is the link to the electronic supplementary material.

Supplementary file1 (PDF 681 kb)

Supplementary file2 (PDF 2323 kb)
