# Supplementary material for: Novel mechanism of drug resistance triggered by tumor-associated macrophages through Heat Shock Factor-1 activation
Source: Cancer Immunol Immunother. 2024 Jan 27;73(2):25. doi: 10.1007/s00262-023-03612-2 (PMC10821977; doi:10.1007/s00262-023-03612-2)
Supplement: Supplementary file 1 — Supplementary file1 (PDF 681 kb) [file 262_2023_3612_MOESM1_ESM.pdf]

Supplementary file to the manuscript “Novel mechanism of drug resistance triggered by tumor-associated macrophages through Heat Shock Factor-1 activation” by Nikotina et al.

For review only

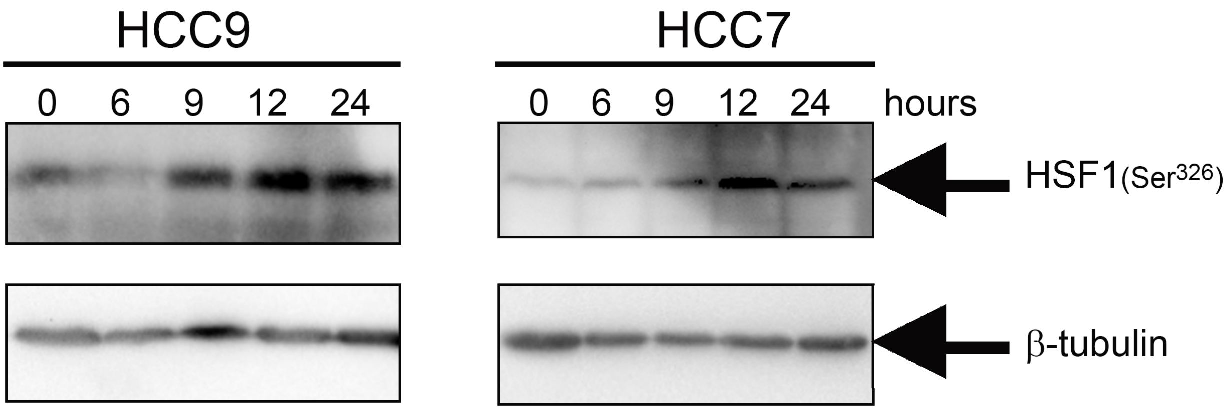

Figure 1S. The dynamics of HSF1 activation due to THP1 cells using the HCC9 and HCC7 cell. HCC6 cells were co-cultured with THP1 at the indicated time intervals and subjected to western blotting. The data showed that the maximum activation of pHSF1^{Ser326} was detected 12 hours after the co-culture of HCC9 and Hcc7 with THP1 and was more than 3-fold higher than the control level.
