# Supplementary material for: Novel mechanism of drug resistance triggered by tumor-associated macrophages through Heat Shock Factor-1 activation
Source: Cancer Immunol Immunother. 2024 Jan 27;73(2):25. doi: 10.1007/s00262-023-03612-2 (PMC10821977; doi:10.1007/s00262-023-03612-2)

Figure 3A

A549 with M0_HSF1

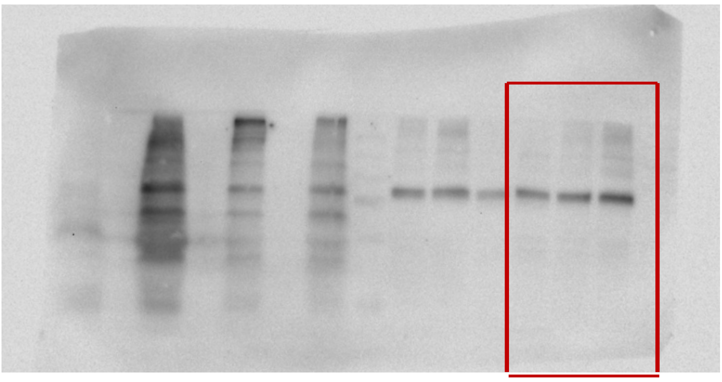

A549 with M0_
Hsp70 and tubulin

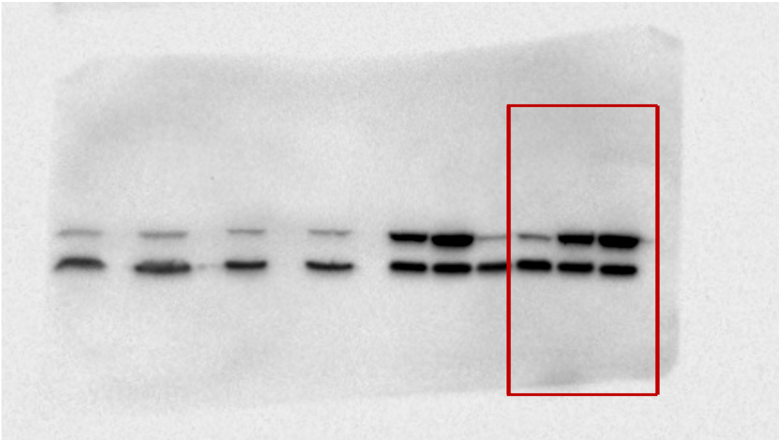

DLD1 with M0 _HSF1

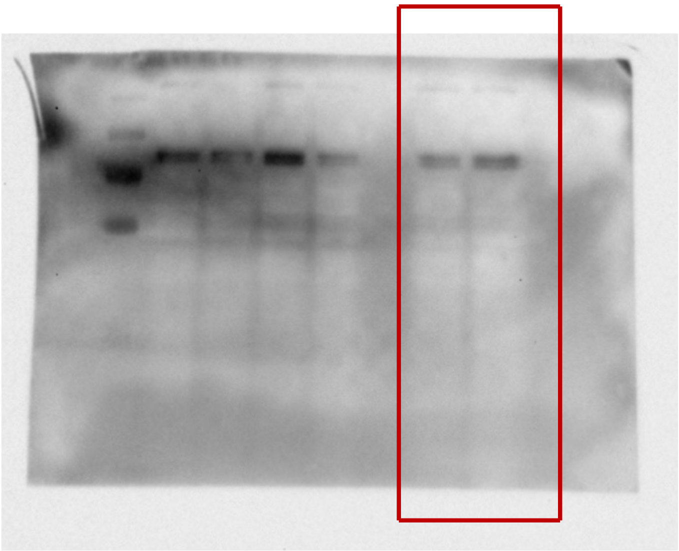

DLD1 with THP1_
Hsp70 and GAPDH

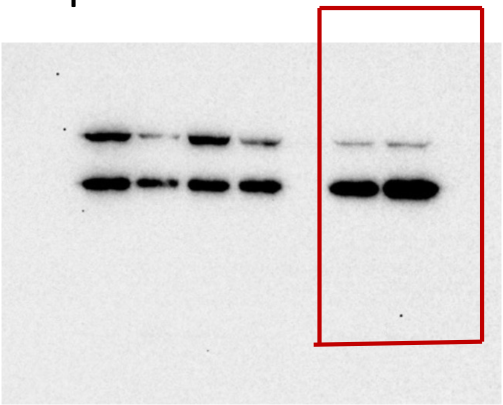

A549 with THP1 _
pHSF1

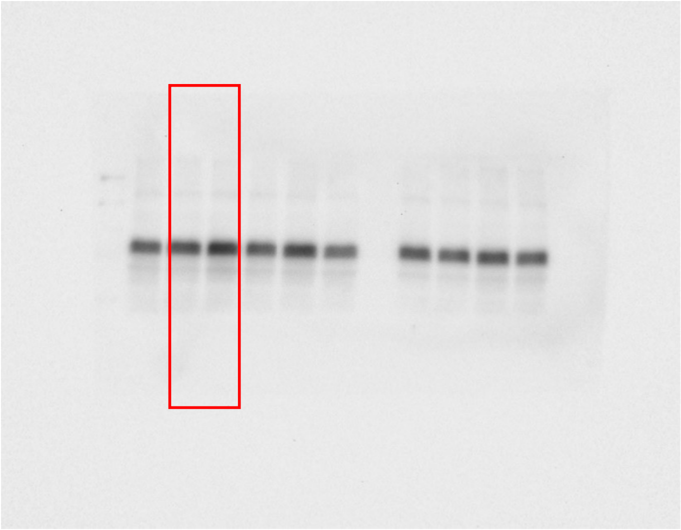

A549 with THP1 _
Hsp70 tub

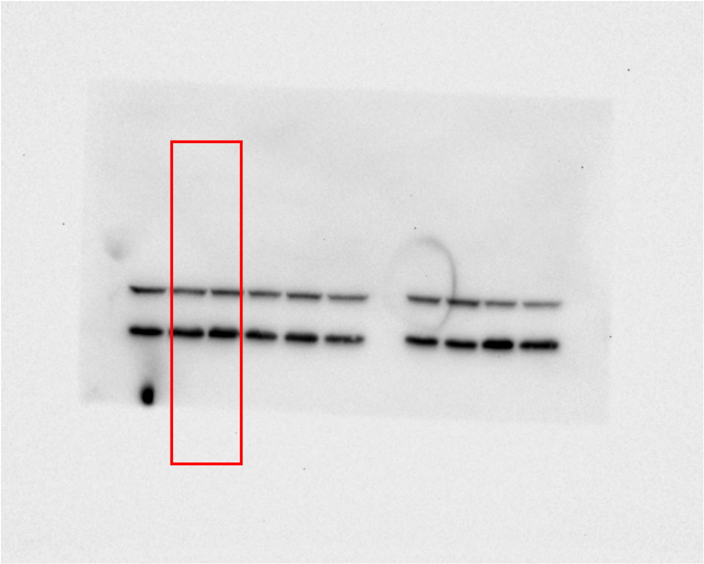

Figure 4A

HCC6 with THP1_ HSF1

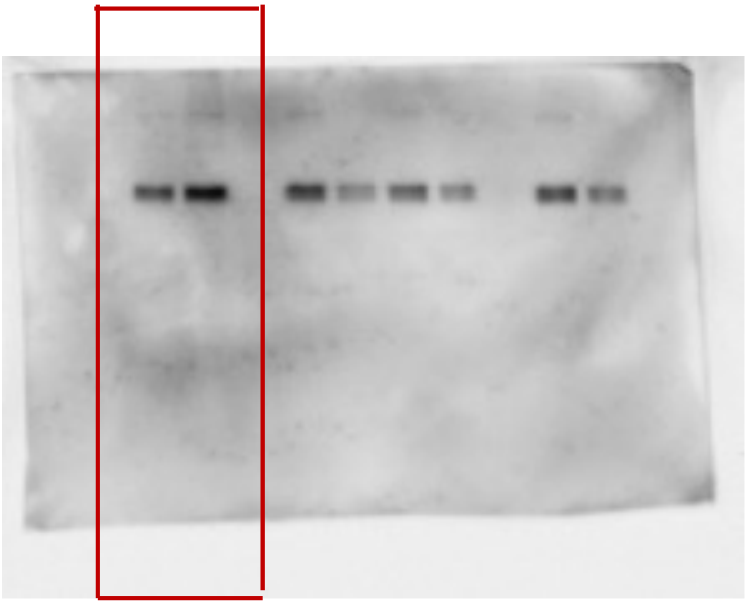

HCC6 with THP1_Hsp70 and ß-tubulin

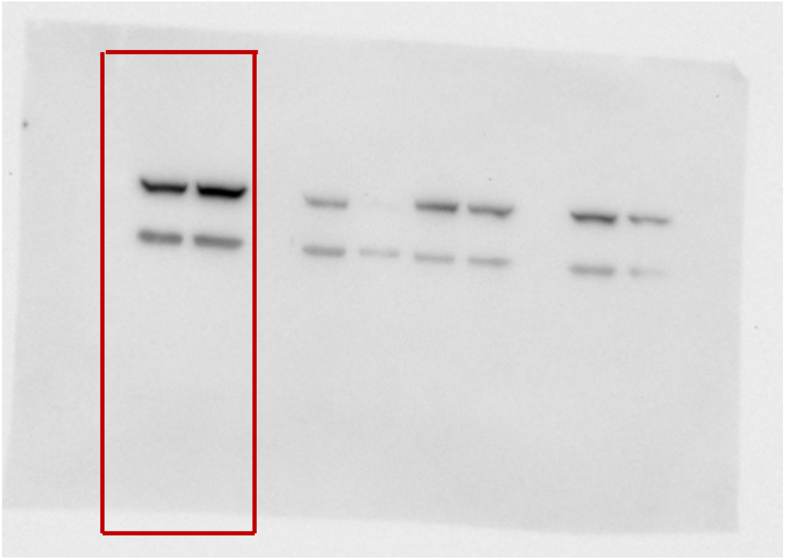

HCC7 with THP1 _ HSF1

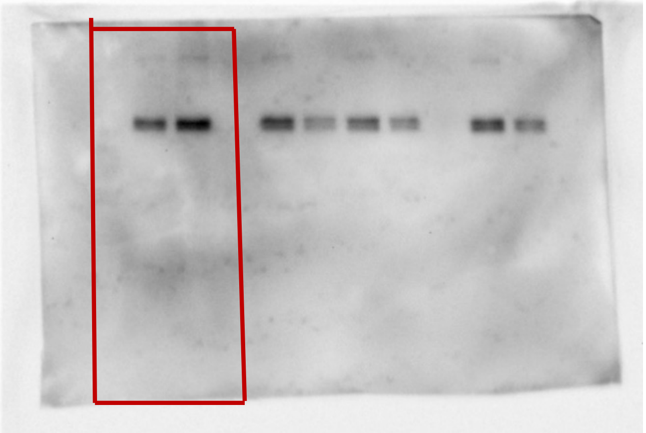

HCC7 with THP1_Hsp70 and ß-tubulin

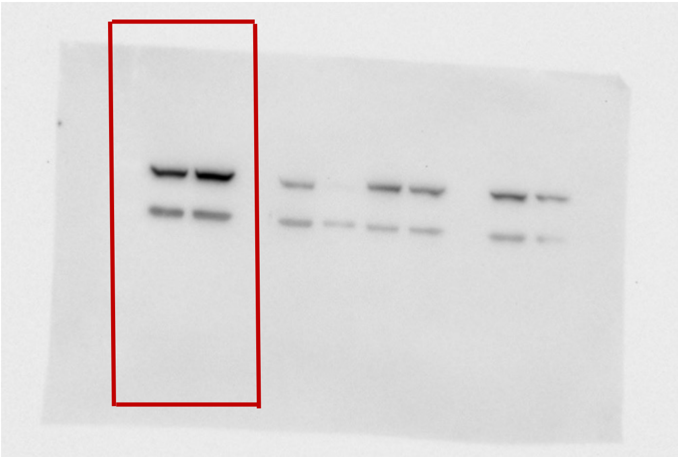

HCC9 with THP1_HSF1

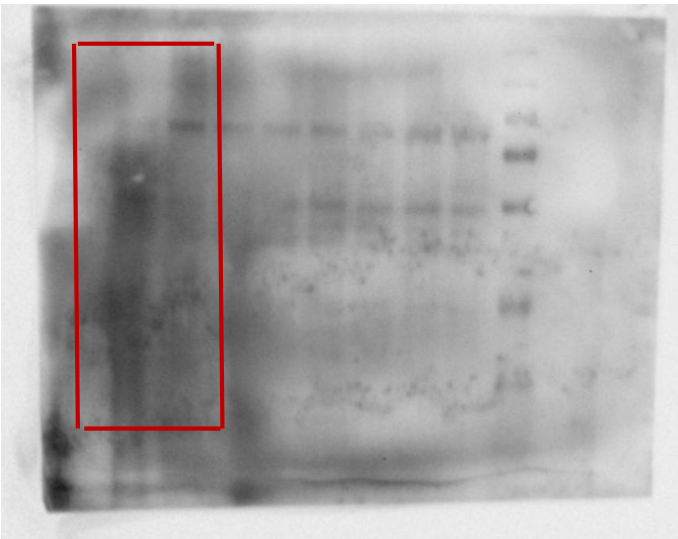

HCC9 with THP1 _Hsp70 and ß-tubulin

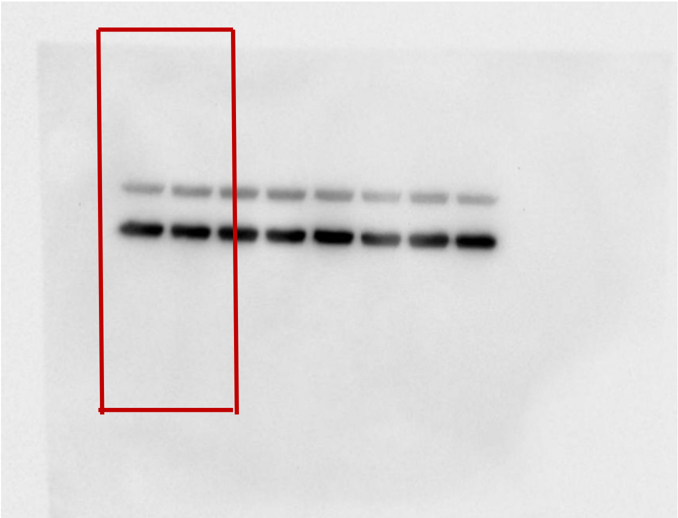

HCCs with M0 _HSF1

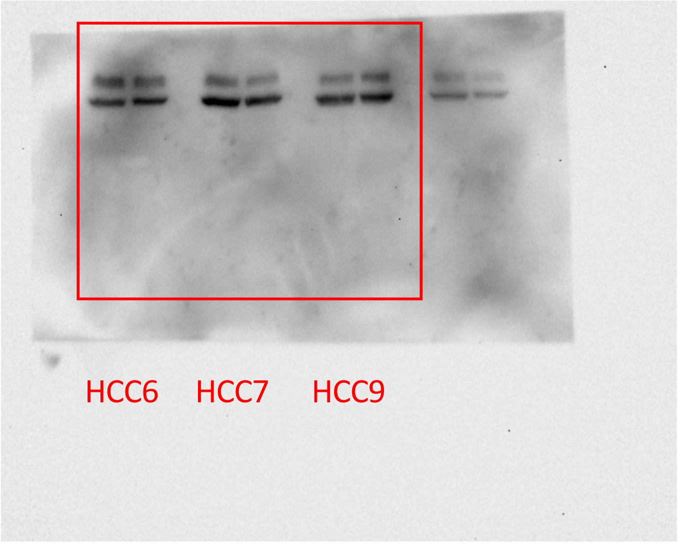

HCCs with M0 _Hsp70 and ß-tubulin

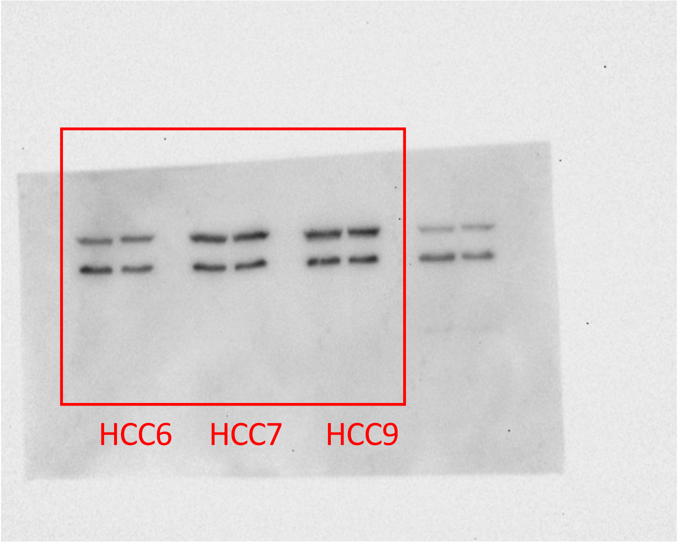

Figure 4C

HCC6 time points _ pHSF1

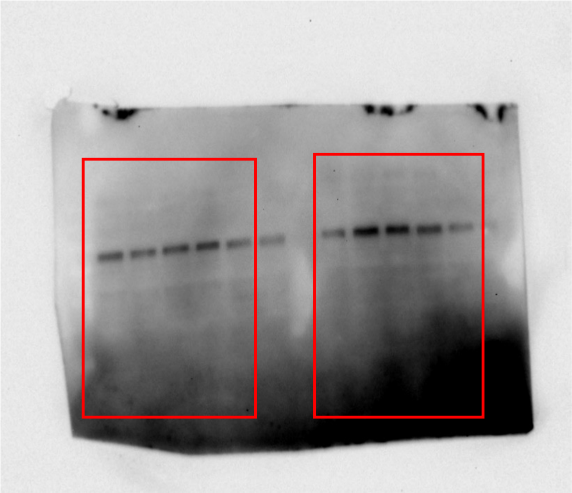

HCC6 time points _ ß-tubulin

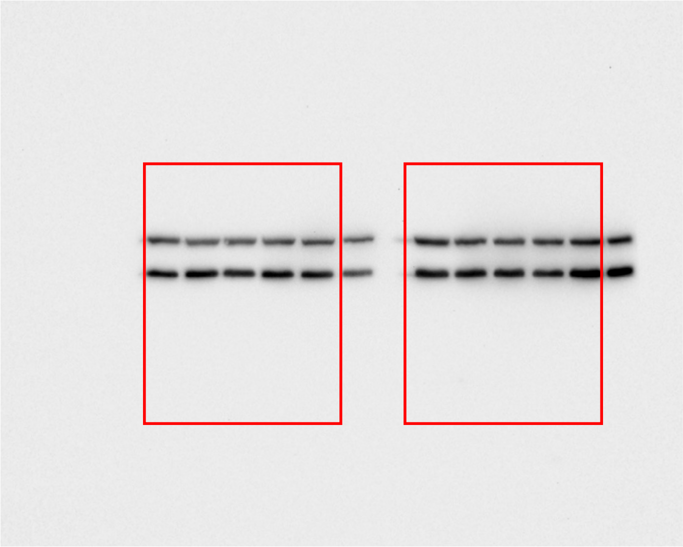

HCC6 time points _ tHSF1

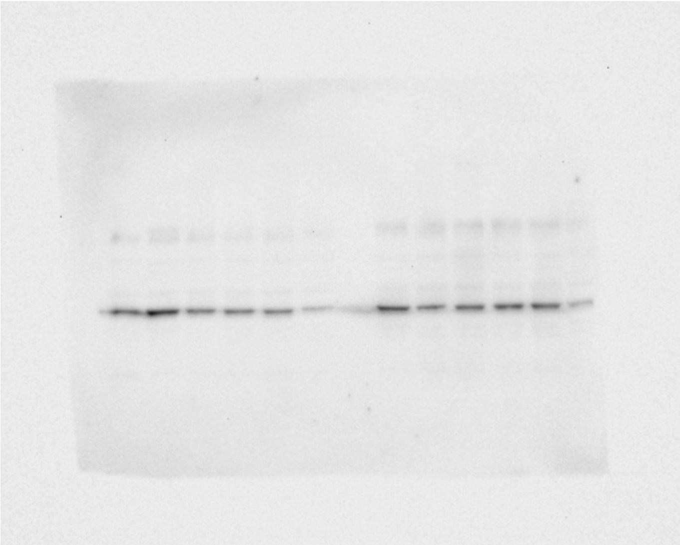

Figure 6A

A549kdHSF1 and A549 scr with THP1 _ pHSF1

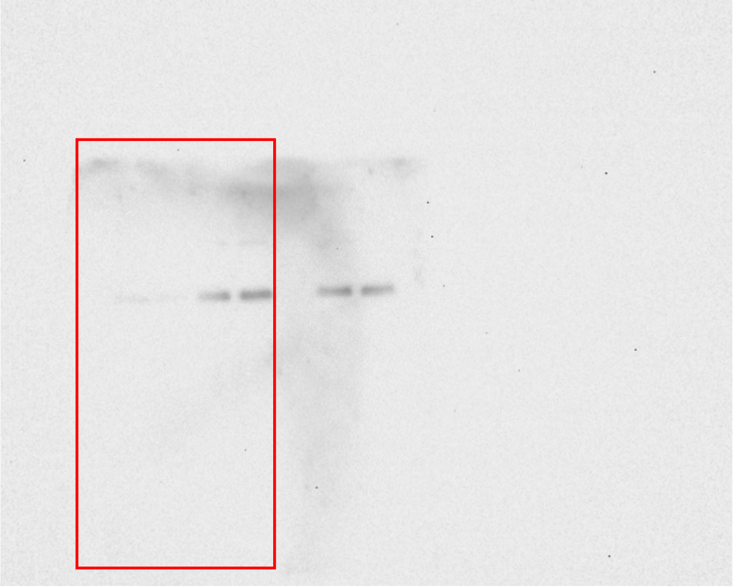

A549kdHSF1 and A549 scr with THP1 _ Hsp70 and ß-tubulin

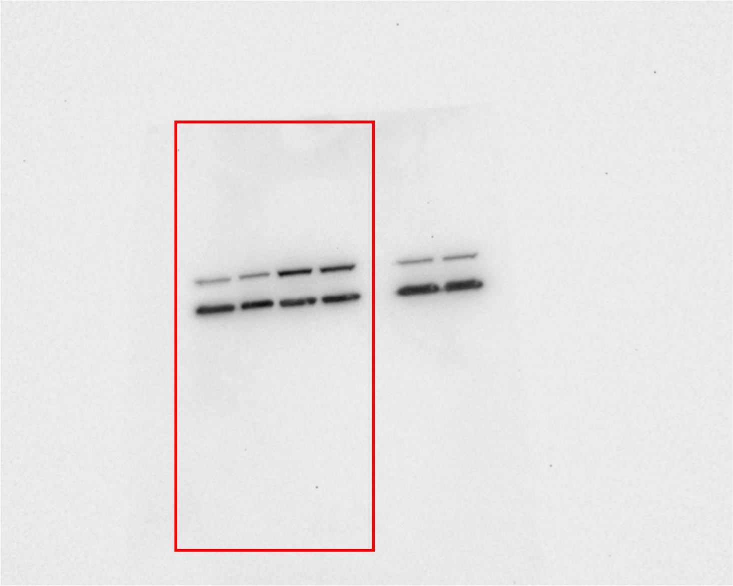

A549kdHSF1 and A549 scr with THP1 _ tHSF1 and ß-tubulin

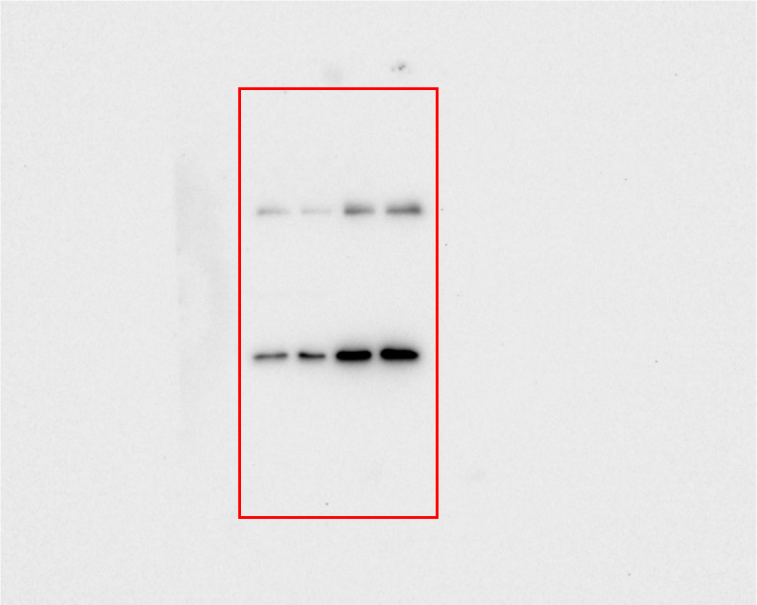

Figure 7A

HCCs with THP1 and CL43 _ Hsp70 and ß-tubulin

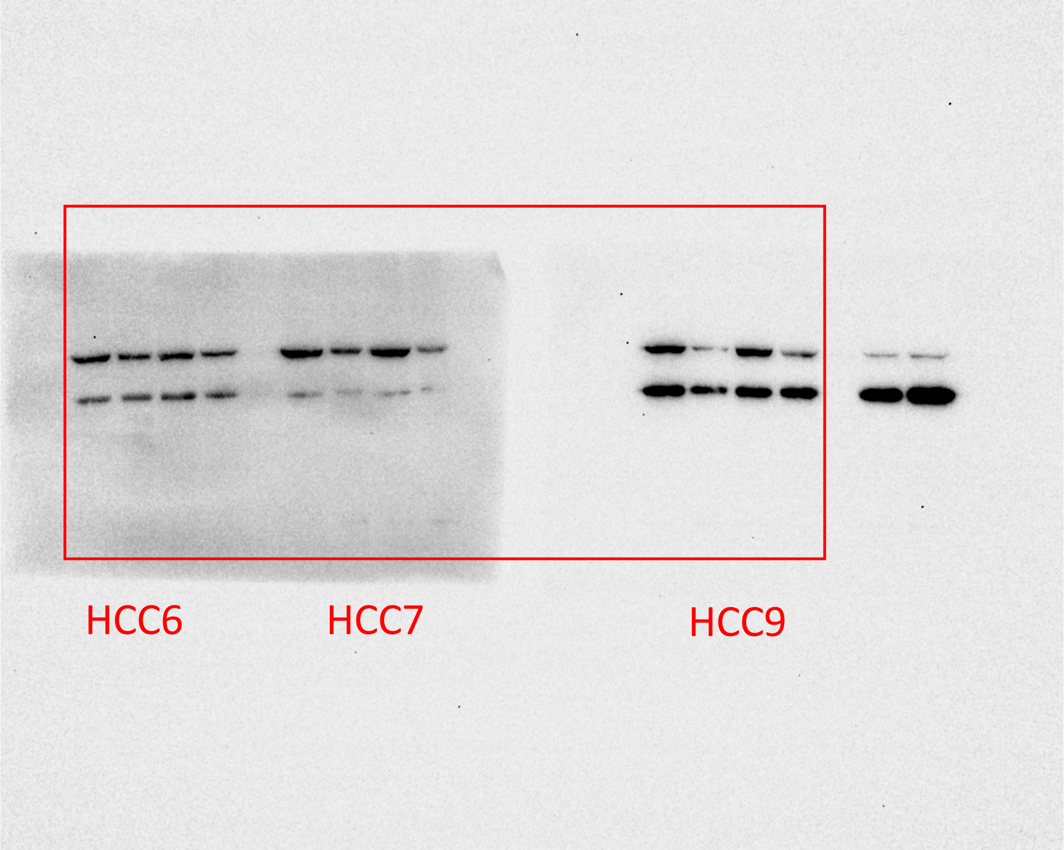

HCC9 with THP1 and CL43 _ pHSF1

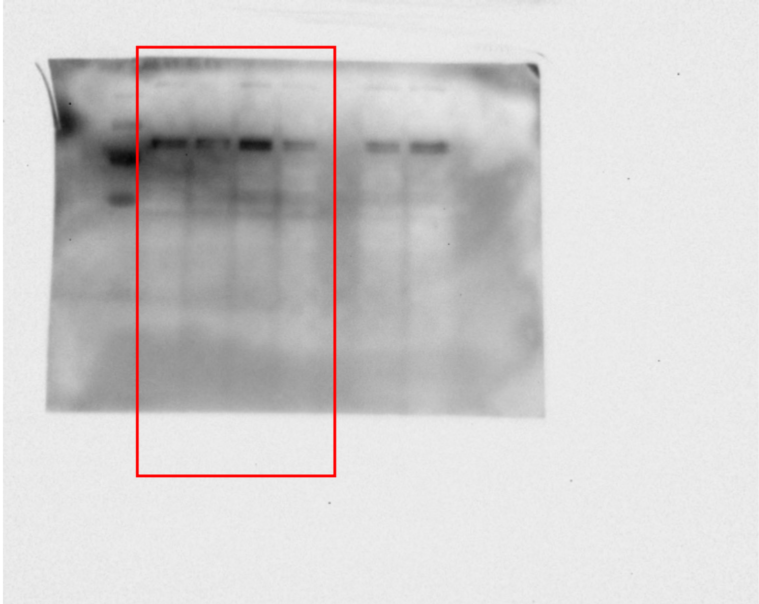

HCC6 and HCC7 with THP1 and CL43 _ pHSF1

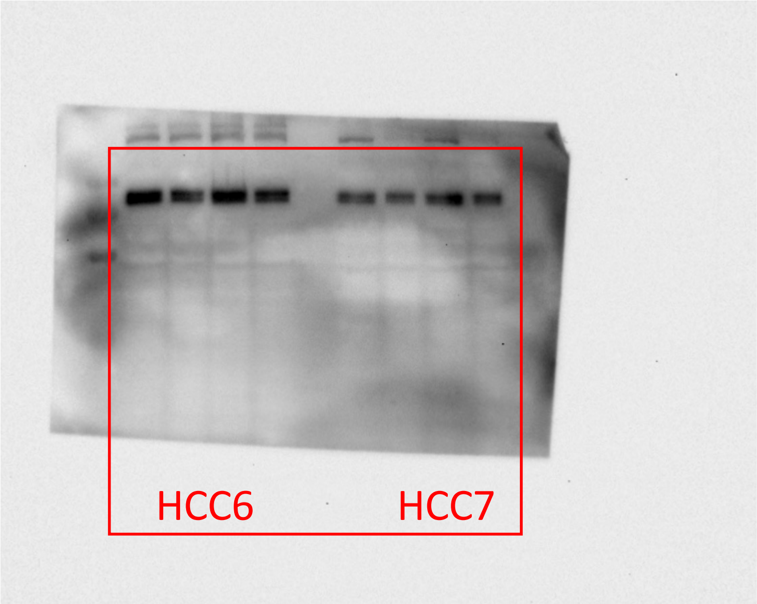

Supplement: Supplementary file 2 — Supplementary file2 (PDF 2323 kb) [file 262_2023_3612_MOESM2_ESM.pdf]
